# Defect engineering of the electronic transport through cuprous oxide interlayers

**DOI:** 10.1038/srep27049

**Published:** 2016-06-03

**Authors:** Mohamed M. Fadlallah, Ulrich Eckern, Udo Schwingenschlögl

**Affiliations:** 1 Institut für Physik, Universität Augsburg, 86135 Augsburg, Germany; 2 Centre for Fundamental Physics, Zewail City of Science and Technology, Giza, Egypt; 3 Physics Department, Faculty of Science, Benha University, Benha, Egypt; 4 King Abdullah University of Science and Technology (KAUST), Physical Science and Engineering Division (PSE), Thuwal 23955-6900, Saudi Arabia

## Abstract

The electronic transport through Au–(Cu_2_O)_*n*_–Au junctions is investigated using first-principles calculations and the nonequilibrium Green’s function method. The effect of varying the thickness (i.e., *n*) is studied as well as that of point defects and anion substitution. For all Cu_2_O thicknesses the conductance is more enhanced by bulk-like (in contrast to near-interface) defects, with the exception of O vacancies and Cl substitutional defects. A similar transmission behavior results from Cu deficiency and N substitution, as well as from Cl substitution and N interstitials for thick Cu_2_O junctions. In agreement with recent experimental observations, it is found that N and Cl doping enhances the conductance. A Frenkel defect, i.e., a superposition of an O interstitial and O substitutional defect, leads to a remarkably high conductance. From the analysis of the defect formation energies, Cu vacancies are found to be particularly stable, in agreement with earlier experimental and theoretical work.

Low production costs and the right band gap are key factors for applications of p-type semiconductors. Cuprous oxide (Cu_2_O) fulfils both criteria, making the material interesting for optoelectronic devices^1^, coatings^2^, sensors^3^, etc. It has a simple cubic structure with lattice constant *a* = 4.27 Å and a direct band gap at the center of the Brillouin zone, experimentally given by 2.17 eV; see ref. 4 for a recent review. From the theoretical point of view, Hartree-Fock and semi-empirical extended Hückel methods have been used for describing the electronic structure, resulting in massive overestimations of the band gap^5^,^6^. On the other hand, density functional theory underestimates the gap, giving 0.5 to 0.7 eV within the local density approximation (LDA)^6^,^7^, 0.99 eV using the LDA + U approach^7^, and 1.08 eV using the self-interaction correction method^8^. The Heyd-Scuseria-Ernzerhof (HSE06) hybrid functional gives a band gap of 2.02 eV, close to the experimental value^7^. All the above mentioned first-principles approaches find the valence band maximum to be dominated by Cu 3*d* states, and the conduction band minimum by Cu 4*s* states, though hybridization is important for the material properties^5^,^6^,^9^.

Vacancies and interstitial defects in Cu_2_O have been studied experimentally for many years, for example, O and Cu vacancies as well as Cu interstitials in refs 10, 11, 12, 13, 14, 15. The effect of doping with nonmetallic anions at the O site has been investigated for Cl^16^ and N^17^,^18^; when doping with Si, Cu vacancy sites are preferably occupied^19^,^20^. Cation doping at the Cu site has been reported for various metals, including Be^21^, Mn^22^, Co^23^, Ni^24^,^25^, Ag^26^, and Cd^27^,^28^. As concerns the theoretical methods, the generalized gradient approximation (GGA) has been employed to investigate the effects of O and Cu vacancies, anti-sites, and interstitials^9^,^29^. The GGA + U approach has been used to study the effects of Mn, Fe, Co, and Ni substitutional doping on the electronic and magnetic properties, indicating the possibility of long-range ferromagnetism when O and Cu vacancies are introduced^30^. GGA calculations also have been reported for doping with the transition metals Ag, Ni, and Zn^31^. Ag substitutional doping turns out to reduce only the band gap, whereas Zn doping leads to n-type conduction.

Since the performance of a device usually is dominated by interface properties^32^,^33^, Cu/Cu_2_O and Au/Cu_2_O interfaces have been addressed in refs 32,34, respectively. In particular, In_2_S_3_/Cu_2_O^35^ and ZnO/Cu_2_O^36^ interfaces have been found to be promising for photovoltaic applications. From the experimental point of view, the electrical resistivities of a Cu_2_O thin film between two gold leads using SiO_2_/Si substrates^37^, and of a sulfur-treated n-type Cu_2_O thin film with Ag, Cu, Au, or Ni front contacts have been measured^38^. However, very little is known about the *theoretical understanding* of electronic transport properties of heterojunctions involving Cu_2_O.

In order to provide insight into this question, we study in the following prototypical Au–(Cu_2_O)_*n*_–Au junctions including different kinds of defects such as vacancies (O or Cu), substitutional, interstitial (N or Cl) and Frenkel defects at different positions in the structures. We carefully optimize the atomic positions, in order to obtain physically relevant results. Note that in the main text our focus is on the optimized structures; but in most figures we include also the results for the unrelaxed structures for comparison, including some brief comments in order to highlight relevant changes due to relaxation. As is apparent from the results, the optimization of the structures strongly affects the electronic structure and transport properties such as the transmission coefficient and the conductance. Generally bulk defects are more effective for enhancing transport than near-interface defects. In order to increase the conductance we suggest that interstitial defects are more suitable than substitutional ones.

We note from the start that formally, one defect for the *n* = 4 junction corresponds to a defect concentration of 3.8%. We also wish to emphasize that the comparison of our model results with experimental data can only be qualitative, since the latter strongly depend on the method of preparation, as well as the concrete substrate—which we are, at least at present, not able to incorporate in a quantitative way.

## Results

Using SIESTA^39^ as electronic structure package, the transport properties are calculated by the SMEAGOL^40^,^41^,^42^ code, which employs the nonequilibrium Green’s functions method. Periodic boundary conditions are applied perpendicular to the [100] transport direction. The Perdew-Burke-Ernzerhof version^43^ of the GGA is utilized for the exchange-correlation potential. Moreover, the energy cutoff is set to 250 Ry, and Monkhorst-Pack k-meshes of 15 × 15 × 25 points for the leads and 15 × 15 × 1 points for the transport calculations are used. Norm-conserving pseudopotentials are employed (fully nonlocal Kleinman-Bylander type^44^ with double-zeta basis set). The pseudopotentials for the Cu, O and Cl atoms include nonlinear core corrections in the exchange correlation potential^45^. The basis set superposition error is corrected using the counterpoise procedure^46^.

A lattice strain of 4% is introduced by assuming an epitaxial interface, and therefore setting the Cu_2_O lattice constant to the Au value of 4.09 Å. All structural optimizations (relaxations) are based on the conjugate gradient method, converging the net force on each atom down to 0.04 eV/Å. The leads are modeled by two atomic layers of face centered cubic [001] Au; in addition, four Au layers on each side of the (Cu_2_O)_*n*_ layer are included in the scattering region. We study Au–(Cu_2_O)_*n*_–Au junctions comprising *n* = 1 to 4 Cu_2_O unit cells, which corresponds to 3 to 9 atomic layers of Cu_2_O.

Comparison to previously published theoretical results on bulk Cu_2_O and Au shows good agreement with our calculations for the bulk materials^7^. The well-known fact that GGA underestimates the *bulk band gap* indeed is an issue of debate, but we take here the point of view that, since the band dispersions are qualitatively correctly described by first-principles calculations, a correct description of *transport through small heterostructures* can be expected, in particular, concerning the defect and doping dependence.

**Figure 1:**
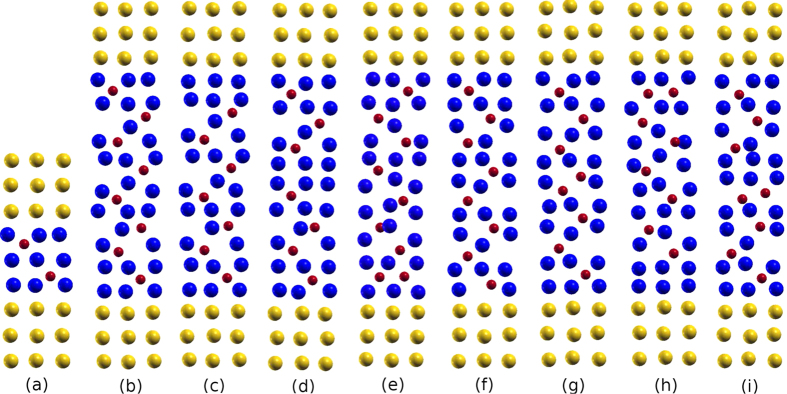
Structurally optimized Au–(Cu_2_O)_*n*_–Au heterojunctions for *n* = 1 (pristine, (**a**)) and *n* = 4 (pristine, (**b**)); O vacancy close to the interface, (**c**); O vacancy in the center, (**d**); interstitial O atom close to the interface, (**e**); Cu vacancy close to the interface, (**f**); Cu vacancy in the center, (**g**); two configurations of Frenkel defects, (**h**), “near-interface”, and (**i**), “bulk-like”. Yellow, blue, and red spheres represent Au, Cu, and O atoms, respectively.

The pristine and defective heterojunctions under study are displayed in Fig. 1. We consider, in particular, defects at or near the interface (near-interface), as well as in the bulk (bulk-like). Concerning O, we study O vacancies near-interface, Fig. 1(c), and bulk-like, Fig. 1(d), and O interstitials near-interface, Fig. 1(e), and in the bulk (not presented in Fig. 1). Furthermore, Cu vacancies at the interface, Fig. 1(f), and in the bulk, Fig. 1(g), are addressed. For Frenkel defects, where an O atom is removed from a certain layer and inserted into another one, there are two possibilities, near-interface, Fig. 1(h), and bulk-like, Fig. 1(i). Finally, N and Cl substitutional and interstitial defects are also considered at different positions in the structure.

Due to the contact between Au and Cu_2_O, the optimization changes the positions of the atoms at the interface and in the bulk of the Cu_2_O interlayer; for pristine junctions, e.g., the Au–Cu distance at the interface is reduced by about 4% due to relaxation. Defects, even those that are two layers away from the interface, alter the atomic positions further: in fact the additional shift of Cu towards Au at the interface is maximal for the cases (e), (g), and (h) in Fig. 1 (another 6%).

The defect formation energies give an indication which defects are more likely to be of experimental relevance than others; it is defined as the energy of the defective structure, minus the energy of the respective “constituents”:


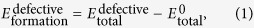


where the subscript “total” refers to the respective total energies. For example, for an X vacancy, 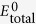 is given by the total energy of the pristine structure, from which the energy of a single X atom is subtracted:


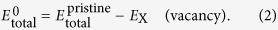


In particular, we determine *E*_Cu_ from the Cu bulk energy, while we assume *E*_O_ to be half the molecular energy. For a substitutional defect, where the atom X is replaced by the atom Y, we have


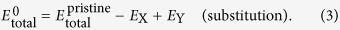


The argumentation for interstitial and Frenkel defects proceeds similarly.

The formation energies—as well as the respective conductances, to be discussed below—are given in Table 1. It is apparent that O interstitials are by far the most stable defects among those considered, followed by Cu vacancy bulk-like, N interstitial bulk-like, and bulk-like Frenkel defects. Generally bulk-like defects are more favorable than interfacial ones except for O vacancies, N interstitial and Cl substitutional defects. The stability of structures with Cu vacancies observed here was noticed in previous theoretical studies^47^,^48^,^49^, and was also seen experimentally^50^,^51^,^52^,^53^.

### Pristine junction

**Figure 2:**
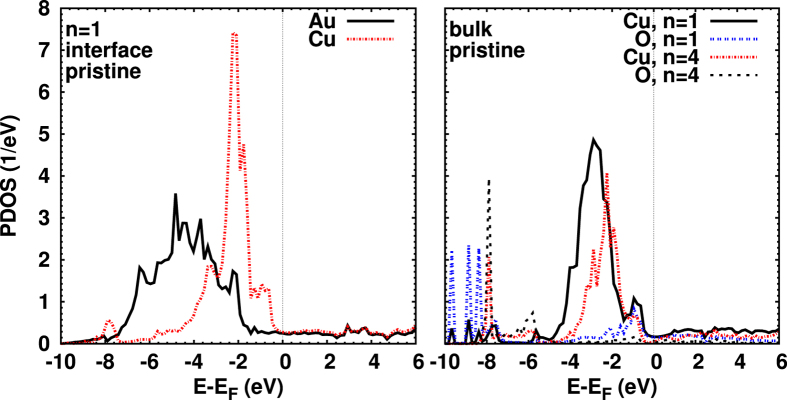
Projected DOS of atoms at the interface (left) and in the center of the Cu_2_O region for *n* = 1 and *n* = 4 (right), after structural relaxation.

The projected density of states (PDOS) of the interfacial atoms for the pristine Au–Cu_2_O–Au heterojunction is shown in Fig. 2 (left). Broad Cu 3*d* and Au 5*d* states are apparent, as well as signs of Cu–Au hybridization, particularly at −2 eV. Figure 2 (right) refers to the bulk-like O and Cu atoms in the center of the Cu_2_O region for *n* = 1 and *n* = 4. The enhanced Cu–O hybridization around −1 eV is characteristic for the *n* = 1 case. Down to about −5 eV we observe the expected dominance of Cu 3*d* states, the DOS structure being broader for *n* = 1.

**Figure 3:**
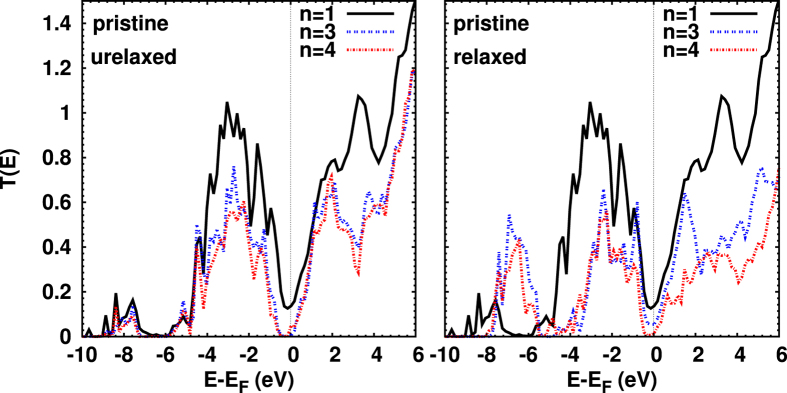
Transmission coefficient for unrelaxed (left) and relaxed (right) heterojunctions. While for the unrelaxed system there is hardly any difference between *n* = 3 and 4, the transport gap appears for the relaxed structure only for *n* = 4. A pronounced transmission around −7 eV appears for the relaxed structure for *n* = 3 and 4, due to significant contributions from Au–Cu interface states (which are absent in the case of *n* = 1). For energies far above *E*_*F*_, the transmission is somewhat reduced upon relaxation for *n* = 3 and 4. On the other hand, hardly any change due to the optimization process is observed for *n* = 1.

The transmission coefficients and thickness (*n*) dependencies are shown in Fig. 3. For the optimized structure (right), *T*(*E*) reflects the shape of the DOS below the Fermi energy, *E*_*F*_, compare Fig. 2: most notable are the peak near −3 eV (due to the localized Cu levels), as well as the almost constant DOS above *E*_*F*_ (due to the extended Cu states). We expect that the transmission at *E*_*F*_ is dominated by Cu states, since Cu is located next to Au in the junctions studied. The corresponding conductance, *G* = *G*_0_*T*(*E*_*F*_), is of the order of 0.15*G*_0_ (for *n* = 1), where *G*_0_ = 2*e*^2^/*h* denotes the conductance quantum (the factor 2 is due to the spin). Increasing *n* to 4, the conductance drops to 0.014*G*_0_.

Above *E*_*F*_, *T*(*E*) increases roughly linearly with energy due to the delocalized Cu 4s states. The transmission decreases with increasing thickness *n*. A transport gap of approximately 0.6 eV appears for the relaxed structure when increasing *n* to 4. For any thickness studied the heterojunction behaves as an n-type conductor. In the following, we omit the results for *n* = 3 since they are similar to those for *n* = 4.

### O and Cu defects

First we discuss the role of O and Cu vacancies and O interstitial defects, cf. Fig. 1(c–g), as well as of Frenkel defects, which consist of an O vacancy and an O interstitial, cf. Fig. 1(h,i).

**Figure 4:**
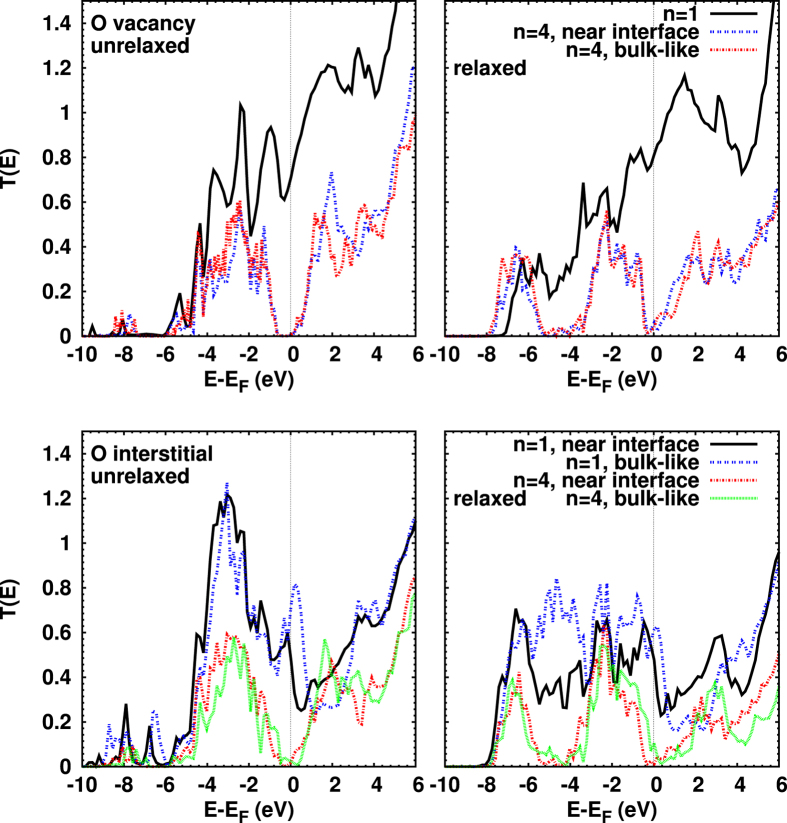
Transmission coefficient for O vacancies (top) and interstitials (bottom), before (left) and after (right) structural optimization; compare Fig. 1(c–e). The pronounced transport gap found for O vacancies (*n* = 4) before relaxation is filled due to structural optimization. On the other hand, there is hardly any relaxation effect for vacancies for *n* = 1, while it appears largest for O interstitial doping. The transmission at *E*_*F*_ generally is higher when the atomic positions are optimized, and that bulk-like defects lead to a higher transmission than near-interface defects.

Figure 4 shows *T*(*E*) for O vacancies (top part of the figure) and O interstitial defects (bottom part of the figure). In the following, we concentrate our discussion on the optimized structures (right). A major change of the transmission, compared to the pristine junction, is observable for both cases for *n* = 1, namely a shift of weight from the peak around −3 eV towards *E*_*F*_, and an enhancement below −5 eV. However, *T*(*E*) is more evenly distributed for an interstitial defect. In addition, *T*(*E*) is enhanced at the Fermi energy by vacancies due to contributions from Au and Cu states, as is apparent from the respective PDOS (not shown here). The conductance is 0.82*G*_0_, and thus considerably larger than in the pristine case.

For the thicker Cu_2_O layer, *n* = 4, we consider O vacancies at the interface and in the center of the Cu_2_O region, see Fig. 1(c,d). The exact position of the vacancy plays only a minor role for *T*(*E*). As compared to the pristine junction, the transport gap at *E*_*F*_ has closed, but the conductance is rather small, about 0.063*G*_0_ and 0.035*G*_0_ for the near-interface and bulk-like vacancy, respectively.

An O interstitial atom in a thin (*n* = 1) Cu_2_O interlayer results in a significant *T*(*E*) down to −8 eV, see Fig. 4 (rightmost). Furthermore, the minimum of *T*(*E*) around *E*_*F*_ is completely washed out, resulting in enhanced conductances of 0.41*G*_0_ (near-interface) and 0.62*G*_0_ (bulk-like). However, the enhancement is slightly less than what is observed for O vacancies. For the thick (*n* = 4) Cu_2_O layer the effect of an interstitial O atom strongly depends on its position: the conductance is considerably higher for the bulk-like than for the near-interface interstitial (0.11*G*_0_ vs. 0.02*G*_0_). This fact can be related to a strong contribution of p orbitals, which lead to a clearly visible peak in the O (bulk-like) PDOS at *E*_*F*_, even larger than the Cu (bulk-like) PDOS. For a near-interface interstitial, on the other hand, this peak is located near −1 eV. Overall *T*(*E*) behaves similar to the pristine junction.

**Figure 5:**
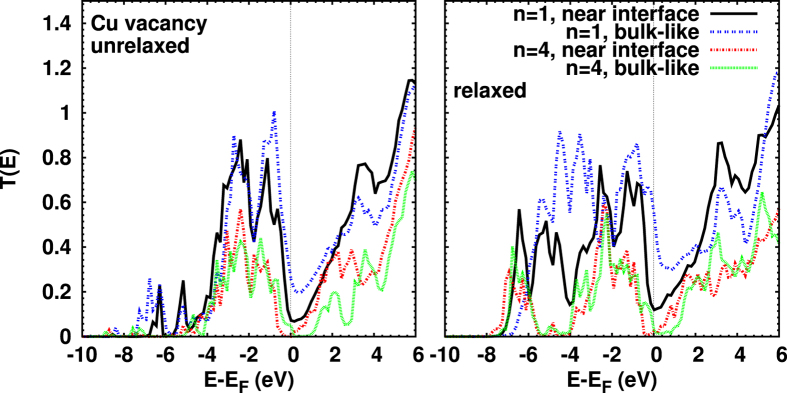
Transmission coefficients for Cu vacancies, before (left) and after (right) structural optimization; compare Fig. 1(f,g). While the overall behavior is similar before and after relaxation, one notes an increase around −7 eV and a decrease for energies far above *E*_*F*_ due to optimization. At *E*_*F*_, the transmission is higher for *n* = 1 compared to *n* = 4 and for a bulk-like compared to near-interface defects. Slightly above *E*_*F*_, a Cu bulk vacancy creates for *n* = 4 a strong transport gap, for both the relaxed and unrelaxed situation.

In Fig. 5 we show the transmission coefficients for Cu vacancies; we focus again the discussion on the optimized structures (right). As compared to the pristine junction, vacancies enhance *T*(*E*) below −5 eV for *n* = 1. For an interfacial Cu vacancy the change in *T*(*E*) below *E*_*F*_ can be related to the sensitivity of the *d* bands to disorder, and the Au(5*d*)–Cu(3*d*) orbital interaction at the interface^54^. The conductance is reduced (compared to the pristine junction) to 0.12*G*_0_ (*n* = 1). For a bulk-like Cu vacancy an enhanced *T*(*E*) is visible between −5 and −4 eV due to contributions from Cu 3d bulk states, and the conductance is given by 0.52*G*_0_. An increase of the conductance due to Cu vacancies is in agreement with previous work on thin films^15^. Independent of its position, a Cu vacancy shifts the minimum of *T*(*E*) around *E*_*F*_ to higher energy.

On the other hand, for a thick (*n* = 4) Cu_2_O layer, the gross shape of *T*(*E*) for Cu vacancies is very similar to the case of O vacancies. The conductance, however, is higher for the bulk-like than for the interfacial Cu vacancy (0.032*G*_0_ vs. 0.026*G*_0_). Note that the conduction type can be modified by Cu vacancies.

**Figure 6:**
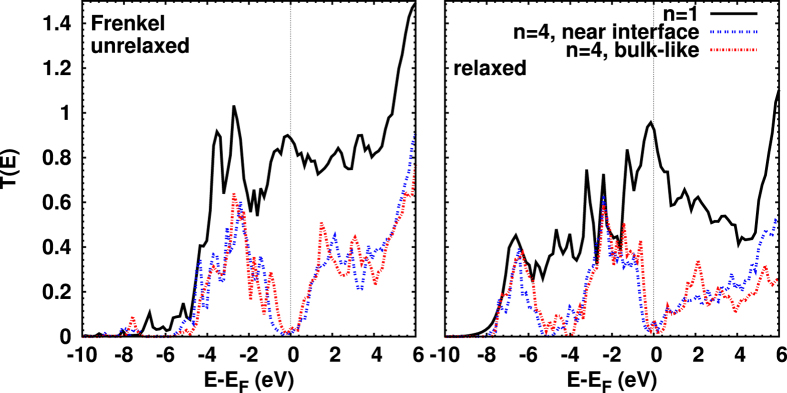
Transmission coefficients for *n* = 1 and 4 heterojunctions with Frenkel defects, before (left) and after (right) structural optimization. The labels “near-interface” and “bulk-like” refer to (h) and (i) in Fig. 1, respectively. For *n* = 1, of course, there is only one type of Frenkel defect. Due to the optimization, there is a drastic change in the transmission in the energy range −6 eV to *E*_*F*_. A pronounced peak near −7 eV appears for all thicknesses. Again an increase of the transmission at *E*_*F*_ is found for *n* = 1 and 4 due to relaxation, while the transmission above *E*_*F*_ is reduced.

Turning to Fig. 6, we observe that a Frenkel defect modifies *T*(*E*) substantially for *n* = 1, leading to a very high conductance of 0.92*G*_0_. For *n* = 4 we consider the two Frenkel defect configurations shown in Fig. 1(h,i). We find no significant difference in *T*(*E*), and obtain essentially the same conductance (0.070 *G*_0_ vs. 0.075*G*_0_) for both of them. Roughly speaking, *T*(*E*) can be considered a combination of the effects of an O vacancy and an O interstitial.

### N and Cl doping

**Figure 7:**
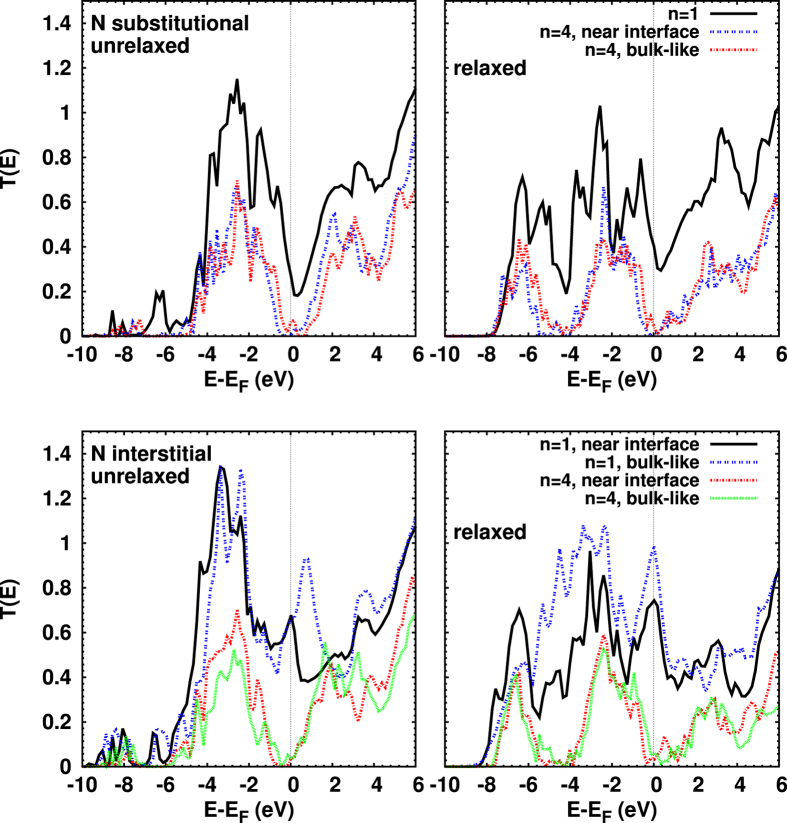
Transmission coefficients for N substitutional (top) and interstitial doping (bottom), before (left) and after (right) structural optimization. For the *n* = 1 heterojunction, besides the appearance of strong transmission around −7 eV as discussed before, the shift of the extrema near the Fermi energy upon relaxation is remarkable, in particular, for N interstitial doping. The transmission is found to depend significantly on the location of the N interstitial only for the optimized case. For *n* = 4, it appears that transmission is slightly higher after relaxation for bulk-like doping (substitutional or interstitial) compared to near-interface doping.

The effect of N substitutional and interstitial doping is addressed in Fig. 7. Clearly the onset of *T*(*E*) is related to the energetic positions of the N relative to the Cu states, and the high *T*(*E*) around −6.5 eV is due to a strong overlap between them. Minima in *T*(*E*), for example near −4 eV, coincide with minima in the Cu PDOS (not shown here). The shift of the minimum of *T*(*E*) around *E*_*F*_ to higher energy is similar to the effect of a Cu vacancy. Conductance values of 0.40*G*_0_, 0.74*G*_0_, and 0.99*G*_0_ are obtained for N substitution, near-interface N interstitial, and bulk-like N interstitial, respectively (*n* = 1). Substitution creates an excess hole, while an interstitial defect creates excess electrons. This explains why the conductance is higher for the interstitial than for the substitutional case. For bulk-like N interstitial there are more transmission channels than for the near-interface N interstitial at *E*_*F*_.

Figure 7 also deals with N doping at different positions for *n* = 4. Comparing substitutional and interstitial doping, the overall energy dependence is quite similar (and similar to the pristine junction), except for subtle differences near *E*_*F*_. As compared to the pristine junction, the transport gap is slightly reduced, and the conductance thus a little enhanced, similarly for bulk-like and near-interface substitution, to about 0.015*G*_0_. Turning to N interstitial defects (*n* = 4), Fig. 7 shows that the bulk-like position of the defect results in a slightly higher conductance than the near-interface position, 0.061*G*_0_ vs. 0.031*G*_0_.

**Figure 8:**
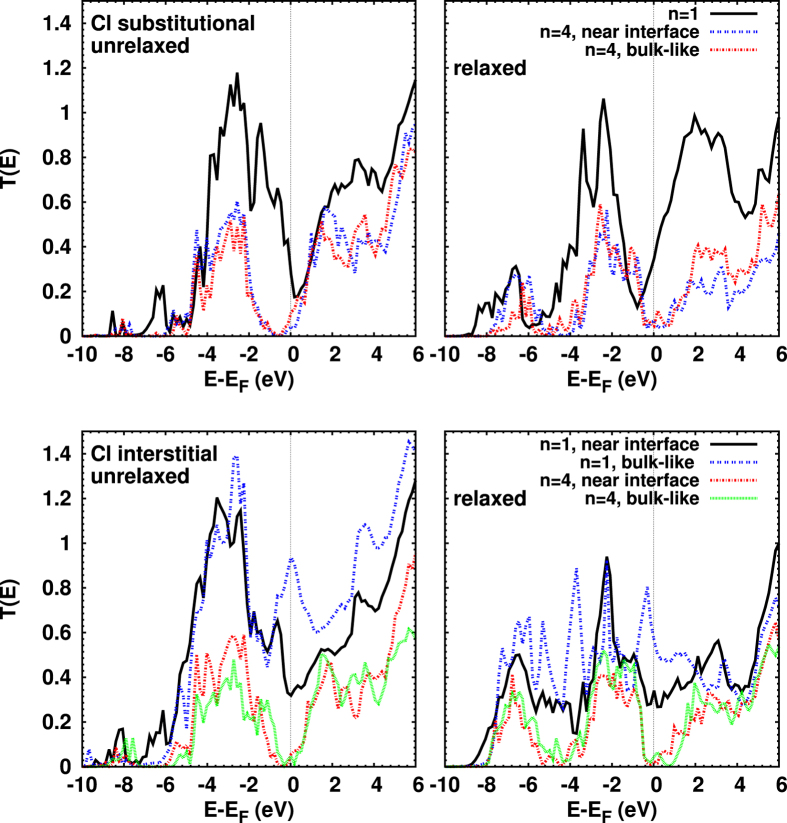
Transmission coefficients for Cl substitutional (top) and interstitial doping (bottom), before (left) and after (right) structural optimization. For substitutional doping, the position of the extrema at *E*_*F*_ for the *n* = 1 unrelaxed system are similar to the N case, compare Fig. 7, whereas the situation is different for *n* = 4. For interstitial doping, on the other hand, for both *n* = 1 and 4 the behavior of the unrelaxed systems is close to the N interstitial. The conductances of the optimized structures are less or equal to the corresponding unoptimized ones for *n* = 1. Again for *n* = 4 the conductances are found to increase upon optimization.

Figure 8 addresses Cl doping. For *n* = 1 substitutional doping, we find close similarities to the pristine junction though the minimum near *E*_*F*_ has shifted to lower energy, which is opposite to what is found for N doping. The conductance of about 0.34*G*_0_ accordingly is higher than in the pristine junction, but lower than in the case of N doping. The shift of the minimum of *T*(*E*) to lower energy appears also for the Cl interstitial defect. A bulk-like Cl interstitial defect leads to a higher conductance (0.56*G*_0_) than a near-interface Cl interstitial defect (0.32*G*_0_).

Interestingly, see Fig. 8, for *n* = 4 we find again a shift of the minimum of *T*(*E*) near *E*_*F*_ to higher energy (compared to the pristine case) for the bulk-like Cl substitutional defect. The conductances are given by 0.065*G*_0_ and 0.043*G*_0_ for near-interface and bulk-like substitution, respectively, which is considerably higher than for N substitution. In contrast to the other cases considered, the Cl bulk-like substitution has a *lower* conductance than the near-interface one. The overall results for Cl interstitials are similar to those for N interstitial defects, but with slightly smaller conductances (0.022*G*_0_ for the near-interface, and 0.033*G*_0_ for the bulk-like case). The calculated conductances for *n* = 4 are summarized in Table 1.

## Discussion

Based on the density functional theory and the nonequilibrium Green’s function approach, a comprehensive study of the electronic structure and transport properties of Au–(Cu_2_O)_*n*_–Au heterojunctions of different thickness, with different point defects, and with different kinds of anion doping of Cu_2_O has been carried out. As to be expected, the transmission decreases with increasing *n*. Compared to the pristine junction, we find for thin interlayers, *n* = 1, for O vacancies and for interstitial O, N, and Cl defects a drastic change of *T*(*E*) around the Fermi energy. Bulk-like defects are found to enhance the transport more effectively than near-interface defects—which appears reasonable since the system has a better chance to adjust to a bulk-like compared to a near-interface disturbance, thereby reducing scattering. Accordingly, we also find that for all cases considered the formation energy is lower for bulk-like than for interface or near-interface disturbances.

Experimentally it has been found that N and Cl doping enhances the conductance^16^,^17^, and that interstitial N defects are more effective than N substitution^18^. While this agrees with our findings, further work is needed to establish the connection between the present model studies and the actual experimental situation, where, in particular, the film thicknesses are much larger than in our model. Note that for Cl we observe the opposite trend: interstitial defects are less effective than substitution. In conclusion, our investigation provides indications on how to improve the electrical and photovoltaic properties of Cu_2_O contacted by two gold leads, namely by appropriate, preferably bulk-like defect engineering. Of course, it must be kept in mind that the detailed experimental conditions may not always be properly reflected by the idealized theoretical modelling.

## Additional Information

**How to cite this article**: Fadlallah, M. M. *et al*. Defect Engineering of the Electronic Transport through Cuprous Oxide Interlayers. *Sci. Rep.* **6**, 27049; doi: 10.1038/srep27049 (2016).

## Figures and Tables

**Table 1:** Formation energies 
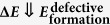
 (in units of eV), and conductances *G* (in units of 10^−2^*G*_0_, *G*_0_ = 2*e*^2^/*h*), for *n* = 4.

	Δ*E*	G									
O vacancy, near interface	0.3	5.8									
O vacancy, bulk-like	0.4	3.2									
Cu vacancy, at interface	−0.3	2.6									
Cu vacancy, bulk-like	−1.6	3.2									
**O**	**Δ*****E***	**G**									
interstitial, near interface	−0.2	2.0									
interstitial, bulk-like	−1.7	11									
Frenkel, near interface	0.8	7.0									
Frenkel, bulk-like	−0.8	7.5									
**N**	**Δ*****E***	**G**									
interstitial, near interface	−0.1	3.1									
interstitial, bulk-like	−1.1	6.1									
substitutional, near interface	2.0	1.5									
substitutional, bulk-like	1.0	1.4									
**Cl**	**Δ*****E***	**G**									
interstitial, near interface	−0.1	2.2									
interstitial, bulk-like	0.2	3.3									
substitutional, near interface	1.1	6.5									
substitutional, bulk-like	1.9	4.3									

For comparison, the conductance of the pristine junction is given by 1.4 × 10^−2^*G*_0_ (*n* = 4): thus the conductance of the defective junctions can be up to a factor of about 8 (for O interstitials, bulk-like) larger than for the “clean” case.

## References

[b1] Zakutayev A., Stevanovic V. & Lany S. Non-equilibrium Alloying controls optoelectronic properties in Cu_2_O thin films for photovoltaic absorber applications. Appl. Phys. Lett. 106, 123903 (2015).

[b2] Nguyen S. H., Lim J.-C. & Lee J.-K. Improving the performance of silicon anode in lithium-ion batteries by Cu_2_O coating layer. J. Appl. Electrochem. 44, 353–360 (2014).

[b3] Deng S. . Reduced Graphene oxide conjugated Cu_2_O nanowire mesocrystals for high-performance NO_2_ gas sensor. J. Am. Chem. Soc. 134, 4905–4917 (2012). 22332949 10.1021/ja211683m

[b4] Meyer B. K. . Binary Copper oxide semiconductors: From materials towards devices. Phys. Status Solidi B 249, 1487–1509 (2012).

[b5] Ruiz E., Alvarez S., Alemany P. & Evarestov R. A. Electronic Structure and properties of Cu_2_O. Phys. Rev. B 56, 7189–7196 (1997).

[b6] Buljan A., Llunell M., Ruiz E. & Alemany P. Color and conductivity in Cu_2_O and CuAlO_2_: A theoretical analysis of d^10^…d^10^ interactions in solid-state compounds. Chem. Mater. 13, 338–344 (2001).

[b7] Heinemann M., Eifert B. & Heiliger C. Band structure and phase stability of the copper oxides Cu_2_O, CuO, and Cu_4_O_3_. Phys. Rev. B 87, 115111 (2013).

[b8] Filippetti A. & Fiorentini V. Coexistence of ionic and metallic bonding in noble-metal oxides. Phys. Rev. B 72, 035128 (2005).

[b9] Nolan M. & Elliott S. D. The p-Type conduction mechanism in Cu_2_O: A first principles study. Phys. Chem. Chem. Phys. 8, 5350–5358 (2006). 19810413 10.1039/b611969g

[b10] Yoshimura M., Revcolevschi A. & Castaing J. Thermogravimetric study of the non-stoichiometry of cuprite Cu_2_O. J. Mater. Sci. 11, 384–386 (1976).

[b11] Xue J. & Dieckmann R. The non-stoichiometry and the point defect structure of cuprous oxide (Cu_2−*δ*_O). J. Phys. Chem. Solids 51, 1263–1275 (1990).

[b12] Porat O. & Riess I. Defect chemistry of Cu_2−*y*_O at elevated temperatures. part i: Non-stoichiometry, phase width and dominant point defects. Solid State Ionics 74, 229–238 (1994).

[b13] Aggarwal S., Topfer J., Tsai T. L. & Dieckmann R. Point defects and transport in binary and ternary, non-stoichiometric oxides. Solid State Ionics 101, 321–331 (1997).

[b14] Kim J. Y., Rodriguez J. A., Hanson J. C., Frenkel A. I. & Lee P. L. Reduction of CuO and Cu_2_O with H_2_: H embedding and kinetic effects in the formation of suboxides. J. Am. Chem. Soc. 125, 10684–10692 (2003). 12940754 10.1021/ja0301673

[b15] Isseroff L. Y. & Carter E. A. Electronic structure of pure and doped cuprous oxide with copper vacancies: Suppression of trap states. Chem. Mater. 25, 253–265 (2013).

[b16] Musa A. O., Akomolafe T. & Carter M. J. Production of cuprous oxide, a solar cell material, by thermal oxidation and a study of its physical and electrical properties. Sol. Energy Mater. Sol. Cells 51, 305–316 (1998).

[b17] Ishizuka S., Kato S., Okamto Y. & Akimoto K. Nitrogen doping into Cu_2_O thin films deposited by reactive radio-frequency magnetron sputtering. Jpn. J. Appl. Phys. 40, 2765–2768 (2001).

[b18] Li J. . Probing defects in nitrogen-doped Cu_2_O. Sci. Rep. 4, 7240 (2014). 25430516 10.1038/srep07240 PMC5384195

[b19] Ishizuka S., Kato S., Maruyama T. & Akimoto K. Control of hole carrier density of polycrystalline Cu_2_O thin films by Si doping. Appl. Phys. Lett. 80, 950–952 (2002).

[b20] Nemoto T. & Nakano T. The effect of silicon on cuprous oxide. Jpn. J. Appl. Phys. 6, 543–544 (1967).

[b21] O’Keeffe M. & Moore W. J. Electrical conductivity of monocrystalline cuprous oxide. J. Chem. Phys. 35, 1324–1328 (1961).

[b22] Wei M. . Room temperature ferromagnetism in bulk Mn-doped Cu_2_O. Appl. Phys. Lett. 86, 072514 (2005).

[b23] Kale S. . Magnetism in cobalt-doped Cu_2_O thin films without and with Al, V, or Zn codopants. App. Phys. Lett. 82, 2100–2102 (2003).

[b24] Kikuchi N. & Tonooka K. Electrical and structural properties of Ni-doped Cu_2_O films prepared by pulsed laser deposition. Thin Solid Films 486, 33–37 (2005).

[b25] Kikuchi N., Tonooka K. & Kusano E. Mechanisms of carrier generation and transport in Ni-doped Cu_2_O. Vacuum 80, 756–760 (2006).

[b26] Tseng C. C., Hsieh J. H., Liu S. J. & Wu W. Effects of Ag contents and deposition temperatures on the electrical and optical behaviors of Ag-doped Cu_2_O thin films. Thin Solid Films 518, 1407–1410 (2009).

[b27] Papadimitriou L., Dimitriadis C. A., Dozsa L. & Andor L. Acceptor states distributed in energy in Cd-doped Cu_2_O. Solid State Commun. 71, 181–185 (1989).

[b28] Papadimitriou L. DLTS evaluation of nonexponential transients of defect levels in cuprous oxide (Cu_2_O). Solid-State Electron. 36, 431–434 (1993).

[b29] Soon A., Cui X. Y., Delley B., Wei S. H. & Stampfl C. Native defect-induced multifarious magnetism in nonstoichiometric cuprous oxide: First-principles study of bulk and surface properties of Cu_2−*δ*_O. Phys. Rev. B 79, 035205 (2009).

[b30] Sieberer M., Redinger J. & Mohn P. Electronic and magnetic structure of cuprous oxide Cu_2_O doped with Mn, Fe, Co, and Ni: A density-functional theory study. Phys. Rev. B 75, 035203 (2007).

[b31] Martinez-Ruiz A., Moreno M. G. & Takeuchi N. First principles calculations of the electronic properties of bulk Cu_2_O, clean and doped with Ag, Ni, and Zn. Solid State Sci. 5, 291–295 (2003).

[b32] Liu D., Zhu Y. F. & Jiang Q. DFT study of CO oxidation on Cu_2_O-Au interfaces at Au-Cu alloy surfaces. RSC Adv. 5, 1587–1597 (2015).

[b33] Zhang L., Kim H. Y. & Henkelman G. CO oxidation at the Au-Cu interface of bimetallic nanoclusters supported on CeO_2_(111). J. Phys. Chem. Lett. 4, 2943–2947 (2013).

[b34] Blajiev O. L. & Hubin A. The effect of silicon on cuprous oxide. Electrochim. Acta 50, 4297–4307 (2005).

[b35] Jayakrishnan R. Photovoltaic response of Cu_2_O/In_2_S3 hetero-structure grown on Cu substrate. Mater. Sci. Semicond. Process. 16, 1608–1612 (2013).

[b36] Yang M., Zhu L., Li Y., Cao L. & Guo Y. Asymmetric interface band alignments of Cu_2_O/ZnO and ZnO/Cu_2_O heterojunctions. J. Alloys Comp. 578, 143–147 (2013).

[b37] De Los Santos Valladares L. . Crystallization and electrical resistivity of Cu_2_O and CuO obtained by thermal oxidation of Cu thin films on SiO_2_/Si substrates. Thin Solid Films 520, 6368–6374 (2012).

[b38] Jayathilaka K. M. D. C., Kapaklis V., Siripala W. & Jayanetti J. K. D. S. Surface treatment of electrodeposited n-type Cu_2_O thin films for applications in Cu_2_O based devices. Phys. Status Solidi RRL 8, 592–595 (2014).

[b39] Soler J. M. . Ab initio derivation of the electronic structure properties across the Cu-Cu_2_O interface. J. Phys.: Condens. Matter 14, 2745–2779 (2002).

[b40] Rocha A. R. . Towards molecular spintronics. Nature Mater. 4, 335–339 (2005). 15750597 10.1038/nmat1349

[b41] Rocha A. R. . Spin and molecular electronics in atomically generated orbital landscapes. Phys. Rev. B 73, 085414 (2006).

[b42] Rungger I. & Sanvito S. Algorithm for the construction of self-energies for electronic transport calculations based on singularity elimination and singular value decomposition. Phys. Rev. B 78, 035407 (2008).

[b43] Perdew J. P., Burke K. & Ernzerhof M. Generalized gradient approximation made simple. Phys. Rev. Lett. 77, 3865–3868 (1996). 10062328 10.1103/PhysRevLett.77.3865

[b44] Kleinman L. & Bylander D. M. Efficacious form for model pseudopotentials. Phys. Rev. Lett. 48, 1425–1428 (1982).

[b45] Louie S. G., Froyen S. & Cohen M. L. Nonlinear ionic pseudopotentials in spin-density-functional calculations. Phys. Rev. B 26, 1738–1742 (1982).

[b46] Boys S. F. & Bernardi F. The calculation of small molecular interactions by the differences of separate total energies. Some procedures with reduced errors. Mol. Phys. 19, 553 (1970).

[b47] Raebiger H., Lany S. & Zunger A. Origins of the p-type nature and cation deficiency in Cu_2_O and related materials. Phys. Rev. B 76, 045209 (2007).

[b48] Scanlon D. O., Morgan B. J. & Watson G. W. Modeling the polaronic nature of p-type defects in Cu_2_O: The failure of GGA and GGA + U. J. Chem. Phys. 131, 124703 (2009). 19791908 10.1063/1.3231869

[b49] Scanlon D. O., Morgan B. J., Watson G. W. & Walsh A. Acceptor levels in p-type Cu_2_O: Rationalizing theory and experiment. Phys. Rev. Lett. 103, 096405 (2009). 19792817 10.1103/PhysRevLett.103.096405

[b50] De Jongh P. E., Vanmaekelbergh D. & Kelly J. J. Photoelectrochemistry of electrodeposited Cu_2_O. J. Electrochem. Soc. 147, 486–489 (2000).

[b51] Garuthara R. & Siripala W. Photoluminescence characterization of polycrystalline n-type Cu_2_O films. J. Lumin. 121, 173–178 (2006).

[b52] Zouaghi M., Tapiero M., Zielinger J. P. & Burgraf R. Hall mobility and hole density in photoactivated Cu_2_O single crystals. Solid State Commun. 8, 1823–1825 (1970).

[b53] Fujinaka M. & Berezin A. A. Cuprous oxide-indium-tin oxide thin film photovoltaic cells. J. Appl. Phys. 54, 3582–3588 (1983).

[b54] Autes G., Barreteau C., Spanjaard D. & Desjonqueres M.-J. Electronic transport in iron atomic contacts: From the infinite wire to realistic geometries. Phys. Rev. B 77, 155437 (2008).

